# Stakeholder-centric participation in large language models enhanced health systems

**DOI:** 10.1038/s44401-025-00024-5

**Published:** 2025-06-18

**Authors:** Zhiyuan Wang, Runze Yan, Sherilyn Francis, Carmen Diaz, Tabor Flickinger, Yufen Lin, Xiao Hu, Laura E. Barnes, Virginia LeBaron

**Affiliations:** 1 School of Engineering and Applied Science, University of Virginia, Charlottesville, VA USA; 2 Nell Hodgson Woodruff School of Nursing, Emory University, Atlanta, GA USA; 3 School of Interactive Computing, Georgia Institute of Technology, Atlanta, GA USA; 4 School of Nursing, University of Virginia, Charlottesville, VA USA; 5 School of Medicine, University of Virginia, Charlottesville, VA USA

**Keywords:** Information technology, Health care, Science, technology and society

## Abstract

Large language models (LLMs) are transforming healthcare by advancing clinical decision support, patient care, and administrative efficiency. However, effectively and sustainably integrating LLMs into healthcare systems requires addressing participatory gaps that may hinder alignment with stakeholders’ practical and ethical needs. This paper explores how participatory methods can be applied throughout the development lifecycle of LLM-enhanced health systems (LLMHS), arguing that: (1) participatory approaches are critical for engaging stakeholders in LLMHS development, and (2) LLM techniques can create novel participatory opportunities that reinforce stakeholder engagement while driving technical innovation in LLMHS. This dual perspective highlights the potential of LLMHS to align technical sophistication with real-world healthcare demands, paving the way for next-generation health systems.

## Introduction

The emergence of Large Language Models (LLMs) has opened transformative possibilities in health systems, showcasing capabilities such as generating human-like text, reasoning, and encoding complex medical domain knowledge^1^. Numerous LLMs, both open-source and proprietary, have been developed globally, with several tailored specifically for healthcare applications, including Med-PaLM, GatorTron, and ClinicalBERT-which support tasks from medical documentation processing^2^ to clinical decision-making^3^ and patient education^4^. In 2023, only 6% of U.S. healthcare systems reported deploying generative AI; however, this figure is projected to grow to 15% by 2024^5^.

Despite these promising capabilities, significant barriers remain in integrating LLMs into healthcare. Many challenges stem from inadequate stakeholder engagement, leading to systems that are not fully aligned with real-world healthcare needs. This misalignment not only undermines trust in system safety and reliability but also results in outputs that are poorly contextualized and ethically or socially problematic^6,7^. lFor example, stakeholder engagement remains unbalanced: while physicians account for 70% of perspectives, essential insights from patients, caregivers, developers, and administrators/policymakers contributed only 11.4%, 7.7%, 7.5%, and 3.4% respectively, across 1721 excerpts from surveyed literature that illustrate a stakeholder’s perspective on clinical AI^8^. Other research indicates that only 5% of healthcare LLM studies (Jan 2022–Feb 2024) used real patient care data^9^, and evaluations have focused predominantly on accuracy rather than on fairness, bias, and toxicity, even though these additional metrics are also important to stakeholders.

Healthcare represents a highly intricate ecosystem, encompassing a diverse array of stakeholders, including patients, clinicians, caregivers, administrators, researchers, and policymakers, each with distinct roles and needs. lTo harness the full potential of LLMs in a healthcare context, it is essential to develop solutions that address the specific requirements and priorities of these stakeholders. Inadequate stakeholder engagement, particularly for health and social care, can misalign research priorities, erode community trust, and undermine research relevance, while limited participation from individuals and communities may exacerbate health disparities^10–12^. Despite some efforts to involve stakeholders, many current development and deployment practices still fall short of the robust stakeholder engagement needed to tackle the ethical, practical, and cultural challenges in healthcare^13^.

**Fig. 1:**
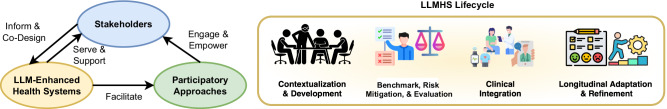
Overview of the triadic relationship among stakeholders, participatory approaches, and LLMHS across the system lifecycle. Participatory approaches engage and empower stakeholders to inform and co-design LLMHS, which in turn better serve and support stakeholders. LLMHS enable and facilitate new participatory opportunities, fostering iterative collaboration across four lifecycle phases: **Contextualization & Development,** **Benchmark, Risk Mitigation, & Evaluation,** **Clinical Integration**, and **Longitudinal Adaptation & Refinement**.

In this paper, we define LLMHS as the use of LLMs to enhance healthcare processes through AI-driven functionalities. We consider participatory approaches^14^ as methodologies that actively engage stakeholders in the design, implementation, and evaluation of LLMHS. We argue that embedding such approaches throughout the LLMHS lifecycle ensures that systems are not only technologically advanced but also ethically sound, practically relevant, and culturally sensitive. More importantly, LLMs can enable new participatory opportunities to enhance stakeholder engagement and healthcare outcomes. We propose a participatory framework (Fig. 1) for co-designing, validating, integrating, and refining LLMHS to ensure they are equitable, practical, and impactful for all stakeholders.

## Preliminaries

### Participatory approaches

Participatory approaches refer to methods that actively involve stakeholders (such as patients, clinicians, and researchers in this context) in the co-design, implementation, and evaluation of systems. Emerging from the social sciences-such as action research^15^ and participatory research^14^-and later adopted by the human-computer interaction community through approaches like participatory design^16^, these methods aim to ensure diverse perspectives foster system relevance and health equity^12,17^. The U.S. Food and Drug Administration’s Collaborative Communities Toolkit^18^ released in 2019, also emphasizes these approaches, advocating for collaborative efforts in the medical devices sector to address healthcare challenges.

In the context of LLMHS, we suggest that participatory approaches are indispensable for fostering stakeholder engagement throughout the entire lifecycle, from initial design to ongoing refinement. Our proposed participatory framework aims to not only highlight how LLMHS can benefit from stakeholder input but also illustrates how the unique capabilities of LLMs can actively enhance participation.

### Participatory gaps of LLMHS

Implementing LLMHS holds great potential, but bridging computational advancements with real-world stakeholder participation remains a key challenge. Here, we outline the key gaps limiting stakeholder engagement:

**Technological limitations:** The growing adoption of LLMs in healthcare faces technological barriers, including high computational demands, energy consumption, and the technical literacy required for deployment^19^. These challenges make LLMs inaccessible to resource-limited healthcare organizations. Additionally, LLMs face issues such as model biases, limited output explainability, and high adaptation costs^20^. In healthcare settings, these limitations are critical; for instance, an LLMHS may generate plausible but incorrect medication recommendations due to LLM hallucinations, eroding clinician trust^21^ and endangering patient well-being. Furthermore, inconsistent outputs from identical clinical prompts and underdeveloped standardization mechanisms raise concerns about reliability and safety^22^.

**Liability concerns:** Liability concerns are a major barrier to adopting LLMHS, highlighting the need for clearer guidelines and collaborative accountability frameworks. Patients worry about who (or what) is accountable for misdiagnoses, treatment errors, data privacy breaches, and technical failures^23^. Healthcare providers face legal uncertainties, particularly when LLMHS recommendations conflict with clinical judgment, creating ambiguity about their liability when overriding AI suggestions^24^. Moreover, emerging evidence indicates that despite the promise of reducing clinician workload, the necessity for meticulous review of LLM-generated content may impose a substantial cognitive and administrative burden on healthcare providers, potentially increasing liability risks^25^.

**Contextualization gaps:** Current LLM systems’ integration into healthcare workflows and critical scenarios remains understudied^26^. Clinicians report frustration with note-taking systems that overlook workflow needs, which reflects gaps in stakeholder input and clinical validation^27^. These gaps are magnified in underserved settings, including historically marginalized, rural, and Indigenous communities. For example, Black and Hispanic stakeholders may face additional challenges such as mistrust in healthcare technologies due to historical inequities, linguistic barriers, and limited access to the digital infrastructure required to support LLMHS^28^. Rural clinics may face significant barriers with cloud-dependent LLMHS, which are often incompatible with limited internet infrastructure^29^, while Indigenous communities can find these systems poorly aligned with traditional healing practices^30^. Engaging diverse stakeholders during design and testing phases is critical for tailoring LLMHS to varied healthcare contexts, improving usability and acceptance^31^.

**Ethical challenges:** Integrating LLMs into healthcare raises fundamental ethical challenges that extend beyond technical and legal barriers. The potential for systematic biases in LLM recommendations that negatively impact marginalized populations, such as racial and gender minorities, underscores the critical need for equitable model development and deployment^32^. Additionally, LLMs may unintentionally erode clinical autonomy by subtly influencing decision-making, especially when their recommendations appear authoritative but lack transparency^22^. This could disempower both clinicians and patients, complicating shared decision-making. Furthermore, the absence of empathetic, human-centered interaction in AI-driven care highlights the importance of preserving compassion in healthcare delivery^23^. To address these ethical concerns, LLM systems must be rigorously validated, designed with fairness and inclusivity at their core, and integrated in ways that complement-rather than replace-human judgment and care.

## Participatory framework in LLMHS lifecycle

As illustrated in Table 1, we identify participatory gaps and suggest solutions throughout each phase of the LLMHS lifecycle.

**Table 1:** Addressing gaps and participatory solutions throughout LLMHS lifecycle

Phase of LLMHS Lifecycle	Key Stakeholders	Participatory Gaps	Participatory Solutions
**Contextualization and Development**	Patients, clinicians, caregivers, and administrators, particularly from underserved groups	Limited stakeholder engagement; systems fail to address diverse needs in workflows and contexts; mistrust in underserved populations	– **LLM-Moderated Co-Design**^35,36^: Accelerates idea generation and iteration through LLM role-play. – **Multi-Agent Simulations**^38^: Simulates diverse scenarios to reveal context-specific challenges. – **Fine-Tuning and RAG**^41,42^: Enhances factuality and contextual relevance through domain-specific adaptation.
**Benchmarking, Risk Mitigation, and Evaluation**	Clinicians, administrators, and policymakers	Lack of transparency and standardization; insufficient real-world clinical data; bias and fairness concerns	– **Stakeholder-Co-Designed Benchmarks**^43^: Establishes evaluation metrics aligned with clinical standards. – **Synthetic Data Generation**^47^: Supplies realistic datasets when real data are scarce. – **Red-Teaming**^49,50^: Uncovers vulnerabilities and mitigates biases via adversarial testing. – **Clinical Trials**^13^: Validates efficacy and safety through controlled, real-world studies.
**Clinical Integration**	Clinicians, patients, caregivers, and administrators	Workflow disruption; trust and liability concerns; lack of transparency and interpretability	– **LLM Agent Workflows**^55^: Streamlines operations with automation while preserving clinician oversight. – **Explainable AI (XAI)**^59^: Enhances transparency and bolsters trust via interpretable decision-making. – **Training Programs**^63^: Boosts role-specific competency and facilitates effective adoption.
**Longitudinal Adaptation and Refinement**	All stakeholders	Limited continuous feedback; systems fail to adapt to evolving clinical practices and stakeholder needs	– **RLHF and Fine-Tuning**^64,66^: Continuously adapts system performance based on ongoing stakeholder feedback. – **Real-Time Monitoring**: Enables proactive adjustments by tracking performance metrics as they occur. – **Agile Development Cycles**^67^: Facilitates iterative and responsive updates aligned with evolving needs. – **Pilot Testing**: Validates system performance through iterative, real-world trials.

*RAG* Retrieval-Augmented Generation, *LLM Agent*, LLM-Empowered Autonomous Agent, *RLHF* Reinforcement Learning with Human Feedback.

### Contextualization and development

The design and development of an LLMHS must prioritize embedding stakeholders’ needs to ensure efficiency and effectiveness, with joint efforts from both technical and healthcare sides. Each stakeholder group, from healthcare professionals to underrepresented community members, brings unique objectives that should guide the system’s contextualization and development.

Stakeholder involvement through co-design and iterative feedback sessions is essential to guarantee both the usability and efficacy of LLMHS. Specifically, these systems should leverage interactive features while promoting health equity across diverse healthcare settings, particularly among individuals of minoritized groups and within medically underserved areas^33^. Incorporating stakeholder-centered methods, such as pre-design surveys and interviews, to identify and define specific healthcare problems early in the process ensures that solutions are not only innovative but also practically grounded. Defining clear “metrics for success”-such as stakeholder satisfaction, reduction in model bias, and improved patient outcomes-can further ensure participatory solutions deliver measurable value. Digital divide issues should be carefully considered, with solutions such as adding offline functionality, supporting low-bandwidth connections, and partnering with local communities to enhance technology access^34^.

#### LLM-moderated participatory co-design

**Fig. 2:**
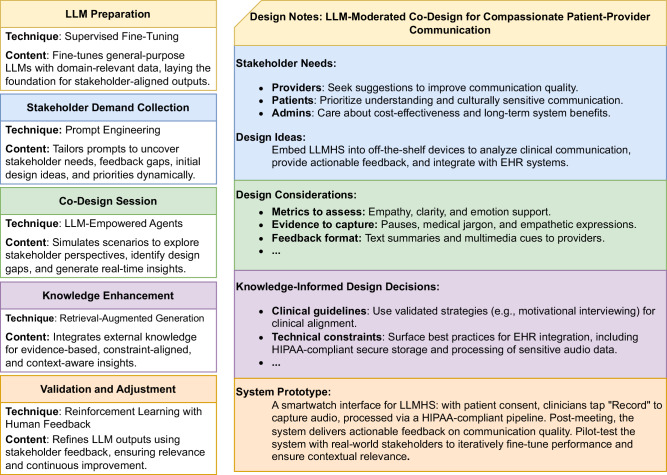
Illustration of an LLM-moderated co-design process for developing the Compassionate Patient-Provider Communication Assessment and Feedback LLMHS. The process integrates stakeholder needs, iterative discussions, and domain knowledge to refine actionable design principles, culminating in a prototype leveraging LLM capabilities and techniques throughout the co-design.

*LLM-Moderated Co-Design*^35^ leverages LLMs as moderators or active participants to guide stakeholder collaboration during design sessions. Figure 2 illustrates the design notes moderated by LLMs for a health system. For example, in co-design workshops, LLMs could simulate application scenarios, generate prototypes from stakeholder input, and pose follow-up questions that deepen discussions. By integrating diverse stakeholder perspectives on system functions, workflows, and target outcomes, the LLM distills these insights into actionable design ideas, drafts mockups, and highlights potential gaps. Recent work^36^ shows that structuring interactions between human stakeholders and LLMs into three phases-Initiation, Discussion, and Convergence-accelerates cross-culture idea generation, reduces facilitator cognitive load, and fosters creativity. Compared to conventional human-driven co-design, this “Human-LLM collaboration” approach can offer more efficient and inclusive participation while preserving human creativity^37^.

Importantly, this adaptive co-design process not only facilitates dynamic stakeholder input but also mitigates practical challenges-such as initial resistance and limited engagement from underserved groups-by leveraging in-context learning and role-play simulations^38^. By simulating diverse stakeholder perspectives, LLMs hold the potential to help narrow participation gaps when certain groups are underrepresented or difficult to involve, making the design process more inclusive and context-aware.

#### Alignment techniques for enhancing relevance and context in co-design

Selectively leveraging advanced LLM techniques improves co-design relevance by dynamically integrating real-time inputs and medical knowledge through a pragmatic, phased approach. LLMs’ *In-Context Learning*^1^ can address workflow challenges without retraining, yielding tailored outputs from existing clinical documentation and standardized workflows. For example, when a clinician identifies medication reconciliation as an issue, the LLM can simulate relevant scenarios and suggest optimized strategies based on available clinical data. l*Chain-of-Thought (CoT) prompting*^39^ breaks down complex design challenges into sequential steps to enhance decision transparency. For instance, in a patient intake system co-design session, the LLM could list key objectives (e.g., reducing wait times), break them into tasks (e.g., streamlining registration, triage, and resource allocation), and propose tailored solutions. This step-by-step process-shown to improve clinical reasoning in differential diagnosis, medical reasoning, and management decision-making^40^-offers stakeholders an actionable roadmap that supports iterative refinement and improved outcomes. Techniques such as *Retrieval-Augmented Generation (RAG)*^41^ and *Model Fine-Tuning*^42^ enhance factuality and relevance by integrating external healthcare and system design literature. For example, in co-design, a hospital might begin by fine-tuning models on their clinical documentation practices, then integrate RAG with standard clinical guidelines, and gradually expand to more complex workflows. This measured expansion helps manage computational requirements while maintaining system reliability and clinical safety. See the Figure for a demonstration of the LLM-moderated co-design process. Notably, organizations can selectively incorporate these modules based on their specific needs and available resources.

### Benchmark, risk mitigation, and evaluation

Rigorous benchmarking, mitigation, and real-world evaluation are critical to ensure ethical deployment and stakeholder trust in LLMHS. Although medical AI holds great promise, research indicates that 43.4% of AI health tools that received regulatory FDA authorization between 2016 and 2022 lacked reported clinical validation data^13^, highlighting the critical need for rigorous evaluation in LLM systems in healthcare .

#### Stakeholder-driven benchmarking and clinical evaluation

*Benchmarking* involves systematically evaluating LLMHS performance against clinical standards or expert judgments to assess system effectiveness and reliability, using metrics like accuracy, robustness, fairness, and computing cost. Specifically, recent study shows that among 519 studies of healthcare LLMs, 95.4% focused on accuracy (e.g., alignment with clinicians’ judgments), 47.0% on comprehensiveness, 18.3% on factuality, 14.8% on robustness, 15.8% on fairness, bias, and toxicity, 4.6% on deployment metrics, and only 1.2% on calibration and uncertainty^43^. For instance, LLM-generated outputs should be compared with those provided by clinicians to evaluate alignment with professional judgment and ensure equitable outcomes. Stakeholder participation should play an active role in co-designing these evaluation metrics, ensuring they not only align with key priorities and patient outcomes but also reflect real-world challenges in clinical workflow^44^. Moreover, it is critical to address the current gap in reporting additional metrics-specifically bias, toxicity, calibration, and deployment considerations these dimensions are essential for stakeholders to guarantee patient safety and equitable care^45,46^. By proactively participating, stakeholders could help shape a benchmark framework assessing both the technical and ethical dimensions of LLM performance in health settings. Particularly, when real-world data are scarce or data collection is prolonged, LLM-enabled synthetic data generation^47^ can produce preliminary datasets that facilitate initial analysis. Acknowledging that synthetic data may oversimplify clinical complexities and introduce biases-and that its use is not feasible in all scenarios-stakeholders’ verification and contextualization of these outputs is crucial. This alternative benchmark method supports preliminary proof-of-concept studies (e.g.,^47,48^) before comprehensive longitudinal evaluations.

#### Collaborative stakeholder engagement for risk and bias mitigation

*Model Mitigation* focuses on managing risks and biases inherent to LLMHS to enhance safety, equity, and inclusion. Red Teaming^49^ is a proactive participatory approach that involves stakeholders in ‘adversarial’ testing scenarios (e.g., presenting ambiguous or conflicting patient data to assess whether the system generates unsafe, biased, or inequitable clinical recommendations) to identify vulnerabilities and failure cases. For example, red-teaming simulations revealed that LLMs were more likely to include pain management for White patients but sometimes omitted it for Black patients, even when clinical cases were identical. Counseling recommendations also reflected bias: the model emphasized empathy for White patients but focused on medicolegal liability for Black patients^50^. As emphasized in interactive machine learning research^51^, integrating human insights is critical to refining system performance. Diverse stakeholder participation fosters participatory auditing, uncovering biases and guiding corrective actions to improve system reliability. However, stakeholders may also hold implicit biases or lack sufficient familiarity with LLMs, which can limit their ability to pinpoint system-generated issues. Addressing these gaps requires targeted stakeholder education and support to clarify LLM-related risks. *Bias Mitigation* methods^52^, such as safety constraints and guardrails (applying rule-based measures to detect and block inappropriate outputs), are essential to ensure equitable outcomes, particularly for marginalized patient populations. In these processes, engaging stakeholders directly informs and enhances the mitigation methods aligned with issues identified, from prompt engineering to model adjustments, ultimately improving system reliability and care quality.

### Clinical integration

Effective clinical integration of LLM-enhanced systems requires embedding these technologies seamlessly into healthcare operations, optimizing engagement among stakeholders and minimizing workflow disruption. Successful implementation depends on three key factors: strategic workflow integration, robust decision support capabilities, and comprehensive stakeholder training^53^. These elements not only ensure efficient adoption but also empower stakeholders to leverage the technology’s full potential in clinical settings.

#### Stakeholder-friendly workflow integration

Current LLM integration into clinical workflows faces significant challenges. For example, LLM-based documentation assistants may inadvertently increase physician cognitive load by requiring extensive verification, particularly in complex cases with multiple comorbidities^54^. When integrated with Electronic Health Records (EHR) systems, LLMs often fail to account for the full clinical context. This can result in incomplete or imprecise interpretations of patient data, which in turn may compromise the quality of clinical decision-making. To address these challenges, implementing a structured feedback mechanism can facilitate continuous system refinement. For example, in medication administration workflows, a staged verification process enables LLMs to identify potential drug interactions and present them in order of severity and urgency. This method allows physicians to quickly review and verify critical issues while integrating with existing clinical protocols and maintaining essential human oversight. Additionally, *Agentic Workflows* (or LLM Autonomous Agents)^55^ show promise in automating multi-step healthcare tasks-such as appointment scheduling^56^, record keeping^57^, and initial diagnostic assessments^58^-to reduce administrative burdens on healthcare providers. However, successful automation depends on establishing clear handoff protocols between AI and clinicians, especially in high-stakes clinical settings. Workflow designs must also include explicit verification points where clinicians can review and adjust AI-generated suggestions, thereby ensuring both efficiency and safety.

#### Transparency and interpretability in supporting stakeholders

Challenges related to transparency extend beyond technical explanations to practical clinical utility. While Explainable AI (XAI)^59^ features provide foundational decision rationales and/or reasoning steps, successful implementation demands stakeholder-specific approaches to explanation delivery that explicitly address current integration failures^60^. For instance, when XAI outputs oversimplify or omit critical uncertainties, they may lack sufficient causability-the ability to convey the underlying rationale-leading clinicians to misinterpret recommendations and ultimately eroding trust in the system^61^. To address these issues, participatory design and iterative feedback are essential. We propose integrating regular feedback sessions into existing clinical infrastructures such as quality improvement meetings-where clinicians and AI ethical experts refine and customize XAI outputs and visualizations for better integration, and patient feedback mechanisms help assess explanation clarity using visualization tools^62^.

#### Training stakeholders for effective clinical adoption

Effective training is crucial for healthcare professionals to fully harness the benefits of LLM systems in clinical settings. While clinicians and administrative staff need training to interpret and apply LLM-generated insights effectively, LLMs themselves can play an active role in this training process. By offering tailored learning modules specific to different roles and simulating healthcare scenarios, LLMs can facilitate hands-on practice that deepens understanding and builds confidence^63^. These training programs foster AI competency across roles, enabling healthcare professionals to leverage LLM capabilities more effectively, thereby improving efficiency and enhancing patient care outcomes.

### Longitudinal adaptation and refinement

Longitudinal adaptation and iteration are pivotal for ensuring sustained efficacy and acceptance among stakeholders. Continuous monitoring and feedback mechanisms should be established to collect usage data and performance metrics, including system accuracy, user behaviors, workflow impact, and patient outcomes. Regular feedback collected through surveys, interviews, focus groups, and automated system interactions will identify areas for refinement. These analytics provide insights into system dynamics, enabling ongoing adaptation to optimize performance as the system evolves.

#### Stakeholder-in-the-loop iterative adaptation

While Reinforcement Learning with Human Feedback (RLHF)^64^ has transformed general LLM development, its adoption in healthcare remains limited due to unique clinical challenges. The inherent complexity of medical decision-making, strict regulatory requirements, and the need for coordinated expert feedback have hindered RLHF’s broader implementation. Recent work shows that an iterative online RLHF approach-where models are continuously updated with fresh human feedback to ensure they are both helpful and harmless-can improve performance without compromising specialized skills^65^. In a robust healthcare RLHF framework, stakeholder-in-the-loop iterative adaptation is essential. Clinical experts can assess clinical validity and evaluate workflow integration, patients and caregivers offer insights on communication clarity and accessibility, and healthcare administrators ensure operational efficiency and regulatory compliance. This multi-stakeholder collaboration can unfold over three phases: initial training-where baseline metrics and feedback protocols are established; refinement-where weighted stakeholder input is iteratively integrated through regular validation cycles; and deployment-where continuous monitoring and systematic updates occur. Additionally, Model Fine-tuning^66^ can complement RLHF by enabling targeted adjustments based on accumulated feedback, ensuring that the system evolves efficiently. Together, these techniques support continuous improvement, maintain clinical safety, and adapt to evolving healthcare needs. Further research into healthcare-specific RLHF metrics and strategies for balancing diverse stakeholder inputs is needed to fully realize the potential of these approaches.

#### Customization and scalability for diverse clinical needs

At the system level, implementing agile development methodologies^67^ can also ensure that the system evolves rapidly in response to stakeholder feedback, while pilot testing and validation studies of new features can identify potential issues before full-scale deployment. This iterative process, combined with stakeholder-driven finetuning and RLHF, ensures that LLMHS remain adaptable, effective, and aligned with clinical needs. System customization and personalization are also important. These adjustments could enable the system to meet the specific needs of different clinical environments, including underrepresented and minority groups. Developing scalable solutions that can adapt to varying resource levels and technological infrastructures is vital for ensuring the system’s broad applicability in diverse healthcare settings.

## Participatory methods in action: two scenarios

To demonstrate how participatory approaches enhance LLMHS development and deployment, we examine two healthcare scenarios where stakeholder engagement is crucial for system success. lThese case studies serve as preliminary empirical illustrations of our proposed framework in action.

### Evaluating and improving healthcare providers’ clinical communication skills

Effective communication between clinicians and patients is critical for trust, satisfaction, and quality health outcomes. Traditional communication evaluation methods that rely on human raters and surveys are resource-intensive and often overlook cultural nuances^68^. In contrast, LLMHS integrated with digital tools offer a scalable solution^48^, enabling real-time analysis of conversations and feedback on key metrics such as empathy and clarity^69,70^ that can help improve the quality of communication. Specifically, in^68^, clinicians and engineers co-designed a smartwatch-based system, CommSense, that can audio-record patient-clinician conversations (with informed consent) and provide actionable feedback to clinicians about their communication. The smartwatch’s embedded microphone records interactions and uses audio models alongside LLMs to extract pre-determined communication metrics (e.g., understanding, empathy, emotion, clarity) and provide feedback to clinicians. For example, CommSense could provide real-time haptic feedback to alert the clinician that excessive medical jargon is being used or that frequent interruptions are occurring, as well as generate post-visit summaries and longitudinal tracking related to communication performance. In our prior work, we quantitatively benchmarked CommSense’s performance using stakeholder-drafted scripts with human annotations^48^. The next phase involves real-world clinical validation and stakeholder interviews to assess its effectiveness in practice.

Furthermore, future work in participatory co-design will aim to more deeply engage healthcare providers in customizing preferred communication metrics across diverse clinical scenarios and aligning system functionality with specific clinical workflows. Incorporating structured feedback loops and RLHF into the iterations of language models and ensuring early stakeholder involvement can help mitigate cultural biases and drive continuous system adaptation. This integration of technical refinement with stakeholder insights demonstrates the potential of the LLMHS framework to improve compassionate healthcare and patients’ quality of life.

### Creating accessible patient educational materials for disadvantaged patients and caregivers

Accessibility of healthcare information presents a significant challenge, particularly for underserved communities facing language barriers and varying health literacy levels^71^. To address this issue, researchers can conduct semi-structured interviews combined with LLMHS-moderated co-design sessions to identify the specific challenges faced by patients and their carers in managing cancer symptoms. These challenges may include difficulties with medical terminology, the need for practical self-care guidance, and a preference for multimedia educational formats. Subsequently, LLMHS can be employed to generate personalized multimedia educational materials-transforming clinical guidelines into accessible visual guides, simplified texts, and interactive elements that reflect the patient’s individual experience and needs^72,73^. Stakeholder-engaged output validation is crucial in this context to ensure content accuracy, cultural alignment, and optimize readability.

## Conclusion and recommendations

To achieve the sustainable and equitable integration of LLMHS, it is essential to embed stakeholder-centric participatory approaches throughout the system lifecycle. Engaging diverse stakeholders through iterative co-design ensures that these systems align with the practical, ethical, and cultural needs of healthcare communities. By addressing critical gaps in transparency, contextualization, and trust, participatory approaches can advance healthcare while fostering broader acceptance among clinicians, patients, and other stakeholders.

The unique capabilities of LLMs themselves offer transformative opportunities for enhancing participatory engagement. Tools such as LLM-moderated co-design and real-time in-context feedback mechanisms enable dynamic collaboration during co-design, while technical foundations like RAG and RLHF ensure systems remain responsive to evolving clinical practices and stakeholder needs. Additionally, integrating XAI components and LLM agentic workflows fosters transparency, mitigates bias, and safeguards clinical autonomy, enhancing trust and usability. These approaches are particularly critical for addressing barriers in underserved and marginalized communities, where culturally sensitive and adaptable solutions are vital.

By operationalizing these strategies, digital health practitioners can design LLMHS that are technically robust, ethically sound, and socially responsible. Moving forward, prioritizing stakeholder-in-the-loop and agile development methodologies with responsible clinical validation will be essential to refine these systems and ensure their integration into diverse healthcare settings advances health equity and fosters trust among stakeholders. Moreover, future work must involve continued validation through well-designed empirical studies and detailed case analyses to further assess and refine the framework’s effectiveness in clinical contexts.

## Data Availability

No datasets were generated or analysed during the current study.
